# Inflammatory Manifestations Associated With Gut Dysbiosis in Alzheimer's Disease

**DOI:** 10.1155/2024/9741811

**Published:** 2024-09-20

**Authors:** Samat Kozhakhmetov, Aiym Kaiyrlykyzy, Zharkyn Jarmukhanov, Elizaveta Vinogradova, Gulnaz Zholdasbekova, Dinara Alzhanova, Jeanette Kunz, Almagul Kushugulova, Sholpan Askarova

**Affiliations:** ^1^ Center for Life Sciences National Laboratory Astana Nazarbayev University, Astana, Kazakhstan; ^2^ Faculty of Medicine and Healthcare al-Farabi Kazakh National University, Almaty, Kazakhstan; ^3^ Institute of Life Sciences Medical University Karaganda, Karagandy, Kazakhstan; ^4^ Department of Neurology Medical University Astana, Astana, Kazakhstan; ^5^ School of Medicine Nazarbayev University, Astana, Kazakhstan

**Keywords:** Alzheimer's disease, cytokines, dysbiosis, gut microbiome, serum adiponectin

## Abstract

Recent studies strongly suggest that gut microbiome can influence brain functions and contribute to the development of Alzheimer's disease (AD). However, reported changes in the gut microbiomes in AD patients from different countries are not similar, and more research is needed to reveal the relationships between human microbiomes and AD in diverse ethnic populations. There is also an assumption that microbiome-associated peripheral inflammation might drive the development of sporadic AD. This cross-sectional study is aimed at analyzing the gut microbial profile and exploring potential associations with blood cytokines and some clinical parameters among individuals diagnosed with Alzheimer's in Kazakhstan. Consistent with previous studies, we have found that the microbial landscape in AD reveals specific alterations in the gut microbiome. Specifically, the AD patient group showed a decreased Firmicutes/Bacteroidetes ratio. The differential abundance analysis highlighted a dysbiosis in the gut microbiota of AD patients, marked by a reduced presence of *Bifidobacterium*, particularly *B. breve*. In our study, AD patients' altered gut microbiota composition notably features an increased presence of Pseudomonadota like *Phyllobacterium* and inflammatory bacteria such as Synergistetes and the Christensenellaceae family. The metabolic profiling of the AD microbiome reveals a predominant presence of pathways related to sugar, carrier molecules, tetrapyrrole, pyrimidine biosynthesis, and nucleic acid processing. This analysis also highlighted a marked reduction in SCFA, carbohydrate, polysaccharide, polyamine, and myo-inositol degradation pathways. The increases in the proinflammatory cytokines IL-1a, IL-8, IL-17A, IL-12p40, TNF-*β*, MCP-1, IL-2, and IL-12p70 and the anti-inflammatory cytokines IL-10 and IL-13 were observed in AD patients. Key variables driving the separation of AD and controls include inflammatory markers (IL-1a and IL-8), growth factors (EGF), lipids (LDL), BMI, and gut microbes, like genus *Tyzzerella* and *Turicibacter* and species *Parabacteroides distasonis* and *Bacteroides eggerthii*. We have also demonstrated that almost all cytokines strongly correlate with serum adiponectin levels and specific microbial taxa in AD patients. Thus, our findings identify potential microbial and inflammatory signatures in an ethnically distinct cohort of AD patients. These could serve as AD biomarkers and microbiota-based therapeutic targets for treating AD.

## 1. Introduction

Alzheimer's disease (AD) is a progressive neurodegenerative disorder that leads to severe memory loss, significant changes in personality and behavior, and an inability to perform routine daily tasks. The prevalence of AD rises with age, affecting approximately 10% of individuals between 65 and 75 years old and 32% of those aged 80 and above [1, 2]. The World Health Organization (WHO) reports a growing incidence of AD annually. Predictions suggest that AD cases could triple by 2050, with the most significant increases occurring in low- and middle-income countries [3]. Several risk factors contribute to the onset of AD. These include nonmodifiable factors such as genetic predisposition and modifiable factors like hypertension during midlife, elevated cholesterol levels, lack of physical activity, obesity, and diabetes. Recent research indicates that gut microbiota may also be a modifiable factor in the onset of sporadic AD [4].

The gut microbiota comprises a diverse, host-specific community of bacteria, archaea, protozoa, fungi, and viruses in the human intestine. This dynamic ecosystem can be shaped by diet, lifestyle, age, gender, and geographic location [4]. Over the past 15 years, the scientific community has taken a heightened interest in gut microbiota due to the established connections between the diversity and composition of the intestinal microbiome and a range of health conditions, including diabetes, obesity, chronic liver disease, allergies, psoriasis, cardiovascular diseases, and cancer. Furthermore, compelling evidence suggests that the gut microbiome plays a role in influencing brain functions [5–7]. Recent studies have demonstrated that the gut microbiota is altered in AD and that these alterations may occur in preclinical AD [8–11].

However, while many studies reported gut dysbiosis in AD, no uniform signature has emerged. Evidence suggests variations in the gut microbiome of AD patients differ across countries [10, 11]. For instance, independent studies conducted at the Alzheimer's Disease Research Center in Wisconsin, United States [10], and Chongqing Medical University in China [11] revealed significant differences in the intestinal microbiome composition of AD patients compared to cognitively healthy peers of the same age, spanning phylum, class, and species levels. However, the specific changes in the gut microbiome of Chinese patients were not entirely congruent with those observed in the United States. These discrepancies might stem from various factors, such as coexisting health conditions, ethnicity, geographical location, lifestyle, and dietary choices [12]. Thus, a deeper exploration is essential to understand the relationship between gut microbiota and AD risk across diverse ethnic groups, which holds significance for developing effective prognostics and preventive strategies.

Another important factor closely related to AD is systemic inflammation [13]. AD patients exhibit neuroinflammation driven by activated microglia, reactive astrocytes, and complement activation. Several proinflammatory molecules involved in central nervous system disorders, such as IL-6, TNF-*α*, and NLRP3, have been identified recently. These molecules are associated with cognitive impairment and pathological hallmarks of AD. This heightened inflammation is also evident in the body fluids of AD patients, notably in the cerebrospinal fluid and blood. In turn, changes in the intestinal microbiota cause an imbalance in host immune regulation, which leads to the development and progression of various infectious and inflammatory diseases [14], and there is an assumption that the development of sporadic AD might be driven by the microbiome-associated peripheral inflammation [15]. It appears that the inflammatory-infectious hypothesis of AD, which has a major role in the gut microbiome, is beginning to gently overshadow the amyloid cascade hypothesis that has dominated for many years.

Nevertheless, the mechanisms connecting peripheral inflammation to neurodegeneration remain elusive. Recent observations suggest that a subset of the gut microbiota drives neuroinflammation in rodents [16]. Evidence also links brain amyloidosis with specific proinflammatory intestinal bacterial taxa and systemic inflammation markers in elderly individuals with cognitive disorders [17]. The results of this study demonstrated that dementia patients with amyloidosis display elevated blood levels of proinflammatory cytokines (IL-6, CXCL2, NLRP3, and IL-1*β*). This elevation coincides with decreased *Eubacterium rectale* and increased *Escherichia/Shigella* in fecal samples. Such observations support the hypothesis that the gut microbiota instigate peripheral inflammation, which in turn could amplify brain amyloidosis, neurodegeneration, and cognitive impairments in AD.

Nevertheless, in-depth research is essential to confirm these associations. As such, this current cross-sectional observational study seeks to examine the gut microbial profile in AD patients from Kazakhstan and probe possible links between the gut microbiota, blood cytokine levels, and specific clinical indicators. This research is a follow-up to our previous pilot study of gut microbiota alterations in patients from Kazakhstan with Alzheimer's dementia [18].

## 2. Materials and Methods

### 2.1. Clinical Assessment

Forty-one individuals diagnosed with Alzheimer's and 42 cognitively normal controls were recruited from Astana City, Kazakhstan's inpatient and outpatient treatment and prevention facilities. Qualified neuropathologists experienced in dementia's clinical diagnosis determined the diagnosis of potential AD. The following criteria were utilized to select AD: (a) the diagnosis of AD-related dementia by the *Diagnostic and Statistical Manual of Mental Disorders* (*DSM-IV*) and the National Institute of Neurological and Communicative Disorders and Stroke, Alzheimer's Disease and Related Disorders Association (NINCDS-ADRDA) criteria; (b) individuals aged 55 years and older at the point of diagnosis and data collection; (c) willingness to voluntarily participate in the study. The subsequent criteria were employed for selecting the control group: (a) the absence of cognitive and memory impairment and (b) willingness to voluntarily participate in the study. The assessment of cognitive functions was conducted using the Mini-Mental State Examination (MMSE) scale for both the study and control groups. We utilized MRI as a supplementary diagnostic tool. MRI provided critical insights into the structural brain changes associated with AD, which significantly supported our clinical evaluations and cognitive assessments. Individuals with severe chronic somatic conditions like kidney and liver diseases, advanced chronic obstructive pulmonary disease, arthritides, and cancer, and those with non-Alzheimer-related mental disorders were excluded from the study. The research protocols were approved by the Ethics Committee of the National Laboratory Astana Nazarbayev University (protocol code 05-2020 dated 24.09.2020), and all participants or their caregivers provided written informed consent for using clinical and genetic information for research purposes.

### 2.2. Fecal Sample Collection, Preprocessing, and Sequencing

Study participants collected feces at home before visiting the clinic using the DaklaPack tubes and Polycool transport pouch collection kit described in our previous study [18]. Fecal samples were collected from participants who self-reported not taking antibiotics within 1 month before sample collection to minimize potential confounding effects on gut microbiota composition. Genomic DNA from fecal samples was extracted using the ZymoBIOMICS DNA Miniprep Kit (Zymo Research, D4300), and sterile *μ*Q water was used as a negative extraction control. OD260/280 Nanodrop and electrophoresis performed a qualitative control of DNA isolation in a 1% agarose gel. The concentration and purity of each DNA sample were determined using an Invitrogen Qubit 3.0 Fluorimeter (Invitrogen, Carlsbad, California, United States). Sterile *μ*Q water served as a negative control. Sequencing was performed on the Illumina NovaSeq 6000 platform at the laboratory of Novogene (Beijing, China) following the standard Illumina protocols [19]. Fecal samples collected from January to August 2020 were sequenced with an average depth of 28.4 million reads.

### 2.3. Blood Sample Collection

Fasting blood samples were collected in plastic sterile single-use vacuum tubes with K2-EDTA (purple cap, 10 mL). For study participants, complete blood count (CBC), glucose, C-reactive protein (CRP), aspartate aminotransferase (AST), alanine aminotransferase (ALT), low-density lipoprotein (LDL), high-density lipoproteins (HDL), total bilirubin (TBIL), and triglycerides (TRIG) were measured. Adiponectin levels in the samples were measured using a quantitative adiponectin determination kit for serum, plasma, and cell culture (Sigma–Aldrich, cat.no: RAB0005) following the manufacturer's protocol.

### 2.4. Multiplex Cytokine Analysis

Multiplex cytokine analysis using Milliplex (MILLIPLEX MAP Human Cytokine/Chemokine Magnetic Bead Panel – Premixed 41 Plex, immunological multiplex analysis HCYTMAG-60K-PX41, Merck) was employed to assess levels of interferon-gamma (IFN-gamma); interferon-alpha (IFN-*α*2); interleukins (IL): IL-1*α*, IL-1*β*, IL-1ra, IL-2, IL-3, IL-4, IL-5, IL-6, IL-7, IL-8, IL-9, IL-10, IL-12 (p40), IL-12 (p70), IL-13, IL-15, IL-17A; tumor necrosis factor-alpha (TNF-alpha); beta (TNF-*β*); monocyte chemoattractant protein-1 (MCP-1); protein-3 (MCP-3); IP-10; MDC (CCL22); MIP-1*α*; MIP-1*β*; PDGF-AA; PDGF-AB/BB; RANTES; sCD40L; EGF; Eotaxin/CCL11; FGF-2; Flt-3 ligand; Fractalkine; G-CSF; GM-CSF; and GRO. Before the study, plasma blood samples were collected from each participant, centrifuged at 3000 rpm for 20 min, and stored at −80°C. The analysis setup procedure was conducted following the manufacturer's protocols. Specifically, 25 *μ*g of plasma (diluted 1:2) were incubated with conjugated antibody-coated magnetic beads for 12 h at 4°C. After washing, bead complexes were incubated with 50 *μ*L of biotinylated detection antibody for 30 min, with the plates being shaken at room temperature. Then, 50 *μ*L of streptavidin-phycoerythrin was added and incubated for 30 min on a plate shaker at (20°C–25°C). After three washes, 100 *μ*L of sheath fluid was added to all examined samples. The bound bead complexes were counted using the FLEXMAP 3D System with the xPONENT 4.0 software.

### 2.5. Data and Statistical Analyses

In this investigation, we analyzed the microbiome of gut samples by focusing on the 16S rRNA gene using the LotuS2 pipeline (Less OTU Scripts 2) [20]. We preprocessed the initial sequencing data to eliminate low-quality reads and filtered out nonbacterial sequences and chimeras. For taxonomic postprocessing of amplicon sequences, we employed SILVA, a 16S rRNA gene database, along with the LCA (last common ancestor) method and UPARSE sequence clustering. Chimeric sequences were identified and removed using the UCHIME algorithm. The remaining high-quality sequences were then grouped into operational taxonomic units (OTUs) at a 97% identity threshold using the LotuS2 pipeline.

To assess the functional capacities of the gut microbiota, we predicted available pathways based on the 16S rRNA sequencing data via PICRUSt2 (phylogenetic investigation of communities by reconstruction of unobserved states) version 2.5.0 with default settings [21]. In this process, we integrated the OTUs into a reference tree encompassing 20,000 complete 16S rRNA sequences from prokaryotic genomes. A reference tree with an NSTI (nearest sequenced taxon index) cutoff of 2 was employed. This reference tree was then employed to estimate each OTU's gene family copy numbers. The forecasted abundance of bacterial metabolic pathways was achieved using the MetaCyc Metabolic Pathway database [22].

Statistical analysis and visualization were performed in Python 3.9.16. Boxplots, violinplots, and scatterplots illustrate differentially abundant features. Additionally, a cladogram was used to represent the hierarchical structure of differentially abundant bacterial taxa using the “LEfSe 1.0.8” library. Alpha diversity was estimated using Shannon, Simpson, and Chao1 indexes. Beta diversity was assessed using Bray–Curtis dissimilarity on Hellinger transformed data. Ordination of beta diversity was performed using principal coordinate analysis (PCoA). ANOSIM and PERMANOVA tests with 9999 permutations were used to assess compositional separation. Alpha and beta diversity calculations and PCoA ordination were performed using the “scikit-bio 0.5.6” library. Differential analysis of taxonomic features was performed using the “PyDESeq2 0.3.5” library (similarly to Vogt et al. [10]), with log2-fold change (LF2C) > 0.5 and adjusted *p* < 0.05 as the significance threshold. Feature importance analysis was performed using the gradient boosting decision trees (GBDT) algorithm using the “LightGBM 3.3.5” library with the importance type set to the “gain” and other hyperparameters set to default. Classification performance was evaluated using leave-one-out (LOO) cross-validation with the area under the precision-recall curve (AUC) as the performance metric. The classification problem was class-balanced. The significance of performance based on identified important features (IF) was assessed using the permutation importance test with 9999 permutations and the square root of dataset length as several stratified folds. Characteristics were considered significant with AUC > 0.5 and *p* < 0.05. Ordination based on essential features was performed using principal component analysis (PCA) after applying z-score normalization. All feature importance calculations were performed with a fixed random seed using the “scikit-learn 1.2.2” library. Correlation analysis was performed using Spearman's rank correlation coefficient exclusively for significantly differentially abundant features using the “SciPy 1.10.1” library. Visualizations were performed using “Matplotlib 3.7.1” and “seaborn 0.11.2” libraries. Where appropriate, two-group comparisons were performed using an independent *T*-test, Welch's *T*-test, or Mann–Whitney *U*-test. A total of 90% confidence intervals (CI) for differences between medians were constructed using the Hodges–Lehmann estimator implemented in SciPy. All calculations involving taxonomic features were performed using features present in at least 25% of the samples. Statistical tests were corrected for multiple comparisons using the Benjamin–Hochberg FDR procedure using the “statsmodels 0.13.5” library.

## 3. Results

### 3.1. Cohort Description and Data Collection

This study involved 83 participants, categorized into two groups: those diagnosed with AD (41 participants) and a control group without cognitive impairment, matched by age and sex (42 participants). The characteristics of these groups are detailed in Table 1.

The mean age for the AD patients was 68.8 years, while that of the control group was 66.5 years; the difference was not statistically significant. In the AD group, the mean MMSE cognitive assessment score was 13.9. Both groups were comparable in age, sex, ethnicity, diabetes status, and cardiovascular disease. However, differences were observed in BMI, serum adiponectin concentrations, TBIL levels, and triglyceride levels.

### 3.2. Dysbiosis of Gut Microbiota in AD Patients

To investigate the gut microbiome of AD patients, we performed 16S rRNA gene sequencing on bacterial DNA extracted from the fecal samples of both AD patients and healthy controls. As mentioned, this research is a follow-up to our previous pilot study of gut microbiota alterations in patients from Kazakhstan with Alzheimer's dementia [18]. We previously analyzed the V1 locus of 16S rDNA. In the present study, we examined the V3-V4 region of 16S rDNA since this region is considered more informative with higher resolution.

**Figure 1:**
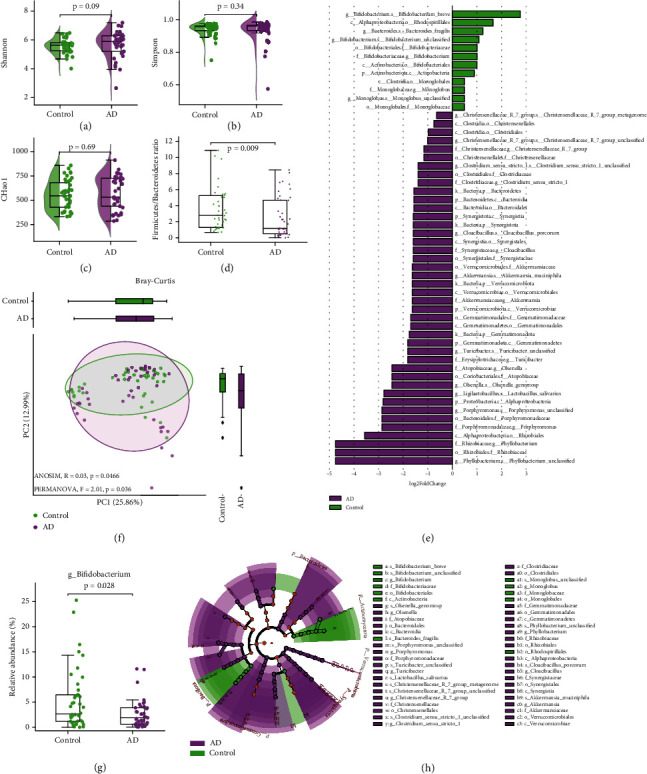
Bacterial community composition in the gut of patients with Alzheimer's disease (AD) compared to controls: (a–c) within-sample diversity of the gut microbiome in AD patients and controls (Mann–Whitney *U* test); (d) boxplot showing the ratio of Firmicutes to Bacteroidetes (F/B) of bacterial phyla in AD and control samples (Mann–Whitney U test); (e) bar plot of log2-fold change in abundance (L2FC) indicating differentially enriched bacterial taxa in the AD group (purple) compared to the control group (green) (calculated using DESeq2 (*p* < 0.05, FDR), plotted using LEfSe); (f) ordination using principal coordinate analysis (PCoA) based on Bray–Curtis dissimilarity showing differences in gut microbial composition between groups (beta diversity), (ANOSIM, *R* = 0.03, *p* = 0.0466; PERMANOVA, *F* = 2.01, *p* = 0.036); (g) bacterial genera overdepleted in the AD group (Mann–Whitney U test); (h) the cladogram represents differentially abundant bacterial taxa; the center of the cladogram represents the kingdom; each subsequent circle is phylogenetically one level lower (phylum, class, order, family, genus, and species). Areas in purple represent taxa enriched in the AD group, and green areas are enriched in the control group. Calculated using DESeq2 (*p* < 0.05, FDR) and plotted using the LEfSe cladogram algorithm. p, phylum; c, class; o, order; f, family; g, genus; s, species.

We have found that AD patients exhibited within-sample diversity comparable to healthy controls. There were no significant differences in diversity as indicated by the Shannon (*p* = 0.09), Simpson (*p* = 0.34), and Chao1 (*p* = 0.69) indices (see Figures 1(a), 1(b), and 1(c)). However, when examining the major bacterial phyla, we observed decreased diversity of both Firmicutes and Bacteroidetes in AD patients (see Figure S1). PCoA based on Bray–Curtis' dissimilarity matrices highlighted distinct microbial compositions between the groups, supported by ANOSIM *R* = 0.03, *p* = 0.0466 and PERMANOVA *F* = 2.01, *p* = 0.036 (see Figure 1(f)). The separation of bacterial communities along PCoA axes was significantly associated (Spearman r, p ≤0.05, FDR) with the enrichment of *Butyricicoccus*, *Ligilactobacillus*, *Clostridium_sensu_stricto_1*, *Turicibacter*, *Enterococcus*, MND1, *Dongia*, RB41, *Skermanella*, *Bryobacter*, *Rhodococcus*, and *Devosia* in AD patients. In contrast, the control group correlated with higher abundances of *Eubacterium hallii*, *Collinsella*, *Catenibacterium*, and Vicinamibacteraceae (see supplement figure 2).

Among study participants, the Firmicutes to Bacteroidetes (F/B) ratio varied from 0.673 to 22.327 with an interquartile range (IQR) of 1.29125–5.19675 and a median value of 2.803 in the control group. In contrast, the AD group's F/B ratio ranged from 0.003 to 8.297, with an IQR of 0.506–4.74 and a median of 1.176. These variations appear to be influenced by a notable rise in the Bacteroidetes phylum's abundance in the AD group, with values ranging from a minimum of 0.059 to a maximum of 65.229, an IQR of 0.158 to 1.187 and a median of 0.399 (MWU *p* = 0.0073; see Figures 1(d) and S1). The observed ratios and variations in the control group align with our earlier pilot report from the Kazakh population, where values ranged from 0.2 to 21, representing typical parameters for healthy individuals [18]. Nonetheless, a lower ratio is often observed in various diseases.

To identify specific taxa associated with the AD group, we analyzed differential OTU abundance using the DESeq2 algorithm. DESeq2 identified a prominent presence of taxa related to gram-negative flora in the AD group. This group displayed an increased abundance of Alphaproteobacteria (genus *Phyllobacterium*), Bacteroidia, bacilli (specifically *Lactobacillus salivarius*), Coriobacteriia, Erysipelotrichia, Gemmatimonadetes, Verrucomicrobiae, Synergistia (genus *Cloacibacillus*), and Clostridia. Conversely, there was a noticeable decrease in Alphaproteobacteria (order Rhodospirillales), Bacteroidia (genus *Bacteroides*), Actinobacteria (genus *Bifidobacterium*), and Clostridia (genus *Monoglobus*). Notably, the AD group showed a pronounced depletion in taxa from the genus *Bifidobacterium*, particularly the species *Bifidobacterium* breve (see Figure 1(g)). This species is recognized for its potential role, possibly through the secretion of exogenous metabolites, in enhancing memory and cognitive abilities in older individuals, as supported by various studies [8, 12]. The L2FC barplot and cladogram in Figures 1(e) and 1(h) visually represent these distinguishing characteristics.

### 3.3. Metabolic Changes in the Bacterial Populations

**Figure 2:**
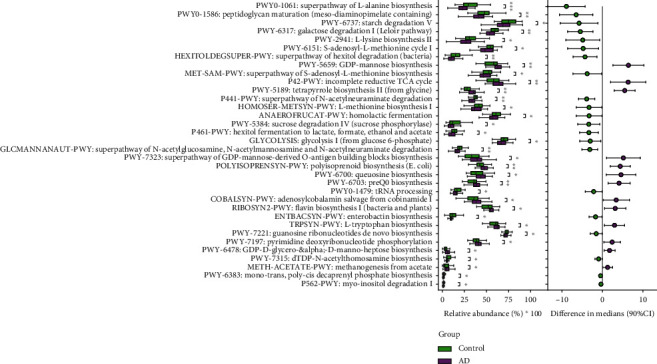
Extended error bar plots showing functional properties that differ between control and Alzheimer's gut microbiomes (*T*-test or Mann–Whitney *U* test, FDR, *p* ≤ 0.05, no 90% CI overlap for difference between medians). The left side shows the relative abundance of metabolic features based on gut microbiome abundance, and the right part visualizes the difference in median abundances between the groups for each feature. Purple indicates the Alzheimer's group; green is the control group. Independent *T*-test, Welch's *T*-test, or Mann–Whitney *U*-test, where appropriate. FDR, *p* ≤ 0.05. 90% confidence intervals (CI) for differences between medians constructed using the Hodges–Lehmann method. The relative abundance is scaled (multiplied by 100).

We assessed metagenomic metabolic pathways using sequencing data and the PICRUSt2 tool with the MetaCyc database to predict metabolic shifts in bacterial populations. Through a comparative analysis employing pairwise comparisons and CI calculations (utilizing either the T-test or Mann–Whitney *U* test, with FDR, *p* ≤ 0.05, and no CI overlap), we identified 34 predicted metabolic pathways with differential relative abundance (see Figure 2). In the AD group, pathways related to sugar, carrier, tetrapyrrole, pyrimidine biosynthesis, and nucleic acid processing were predicted to be relatively more abundant. In contrast, the control group exhibited a more excellent predicted representation in amino acid biosynthesis, enterobactin, and purine pathways. When comparing the predicted metabolic profiles of the AD group's gut microbiome to the control group, there was a reduced predicted representation in pathways such as short-chain fatty acid (SCFA) fermentation, carbohydrates, polysaccharides, polyamines, and myo-inositol degradation.

### 3.4. Cytokine Features of Alzheimer's Patients

**Figure 3:**
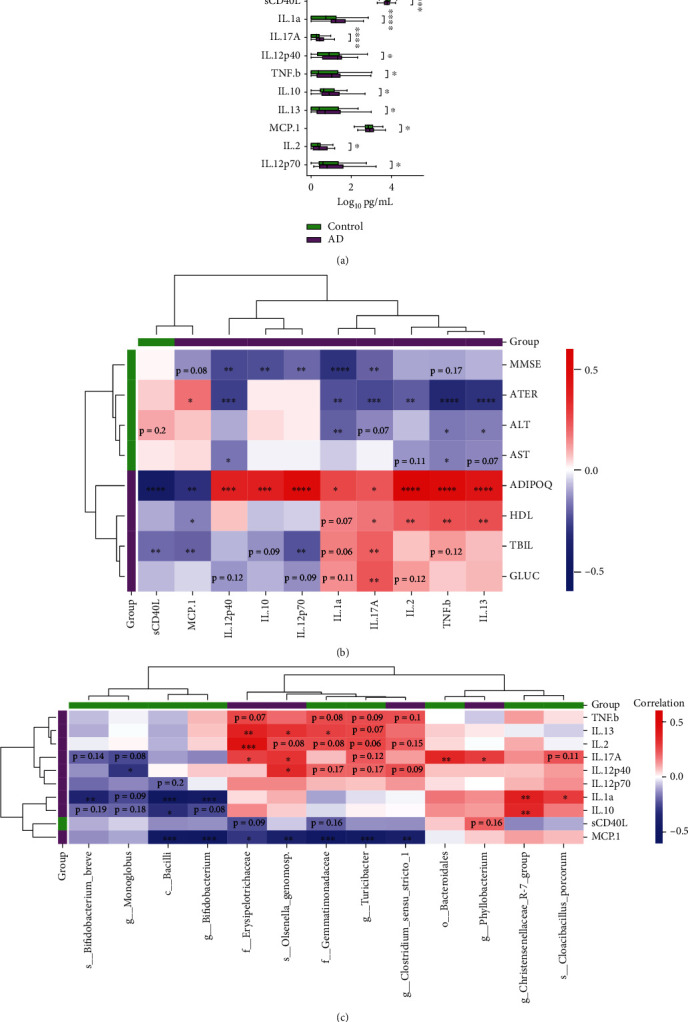
Cytokine expression profiles in Alzheimer's disease patients (AD) and controls: (a) cytokine levels differ between the AD group (purple) and the control (green) (Mann–Whitney U test, FDR, *p* ≤ 0.05); (b, c) correlation analyses between clinical variables and immunological parameters and significantly differentially abundant taxa identified in DESeq2 analysis and immunological parameters. On correlation cluster-grams - parameters enriched in AD are highlighted in purple, and control is highlighted in green on the sides of a heatmap. Red indicates a positive correlation; blue indicates a negative correlation. Spearman *r*, FDR, 0.05. ^∗^*p* ≤ 0.05, ^∗∗^*p* ≤ 0.01, ^∗∗∗^*p* ≤ 0.001, ^∗∗∗∗^*p* ≤ 0.0001.

Previous studies have shown a link between changes in cytokine expression and the development of AD [23]. Comparison of the production levels of pro and anti-inflammatory cytokines in serum showed a significant increase in the expression of the proinflammatory cytokines: IL-1a, IL-17A, IL-12p40, TNF-b, MCP-1, IL-2, and IL-12p70, and the anti-inflammatory cytokines: IL-10, IL-13, and depletion of sCD40Lin AD patients (see Figure 3(a)).

Examination of the relationships between serum cytokine levels and clinical parameters among AD patients and controls revealed a strong positive correlation between adiponectin (ADIPOQ) and all cytokines, with the exceptions of the transmembrane glycoprotein sCD40L and the chemokine MCP1 (see Figure 3(b)). There was a positive association between IL-17A and HDL, TBIL, and GLUC. However, IL-17A displayed negative correlations with MMSE, ATER, and ALT, which were elevated in the control group. IL-17A, IL-2, IL-13, and TNF-*β* all had positive correlations with HDL, whereas MCP1 had a negative correlation. A positive correlation with IL-17A was observed concerning TBIL, and negative correlations were noted with sCD40L, MCP1, and IL-12p70.

Further, when evaluating the relationships between significantly elevated cytokines and bacterial taxa, IL-13 showed positive correlations with Erysipelotrichaceae, Olsenella genomosp, and Gemmatimonadaceae. Both IL-2 and IL-17A were positively correlated with Erysipelotrichaceae. Moreover, IL-17A had positive associations with Bacteroidales and *Phyllobacterium*. IL-1*α* correlates positively with Christensenellaceae R-7 and Cloacibacillus porcorum but negatively with *Bifidobacterium* and Bacilli. Lastly, IL-10 was positively correlated with the Christensenellaceae R-7 group and negatively correlated with Bacilli (Figure 3(c)).

### 3.5. The Key Variables Differentiating AD Patients From Controls

**Figure 4:**
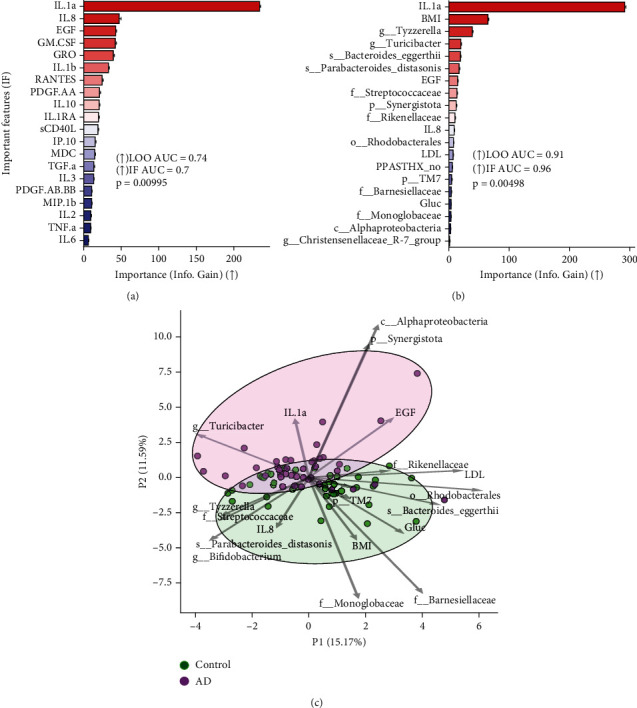
Feature importance analysis for discriminating between Alzheimer's disease (AD) patients and controls: (a) the importance bar plot shows the 20 most important blood inflammatory markers, the top discriminators between the AD and control groups; (b) the importance bar plot shows the top 20 discriminators between the AD and control groups; the horizontal axis represents the measured effect of the feature on discrimination (“importance”). Feature names ranked by importance (↑) are shown on the vertical axis. (c) principal component analysis (PCA) ordination based on the most important z score normalized discriminative features. PCA loadings are shown as arrows; the length of the arrow indicates the relative effect of the feature on the ordination, and its angle indicates the direction of the impact. Feature importance analysis was performed using the gradient boosting decision trees (GBDT) algorithm with performance evaluation using leave-one-out (LOO) cross-validation with the area under the precision-recall curve (AUC) as the performance metric. The significance of performance based on identified important features (IF) was assessed using a permutation importance test with 9999 permutations. ADIPOQ was excluded from the analysis.

Figure 4 shows the key variables differentiating AD patients from controls in the GBDT model. Inflammatory markers like IL-1a, IL-8, and EGF (see Figure 4(a)) are among the most critical discriminators, along with other factors like BMI, LDL, and microbial taxa (see Figure 4(b)). The GBDT model with these critical features achieves high discrimination between AD and controls (IF AUC = 0.96), which is better than using all features (LOO AUC = 0.91). Key variables driving the separation of AD and controls include inflammatory markers (IL-1a, IL-8), growth factors (EGF), lipids (LDL), BMI, and gut microbes, like genus *Tyzzerella* and *Turicibacter*, and species *Parabacteroides distasonis* and *Bacteroides eggerthii* (see Figure 4(c)).

## 4. Discussion

Our study of the microbial landscape in AD reveals specific alterations in the gut microbiome. While alpha diversity measures, as represented by the Shannon, Simpson, and Chao1 indices, did not show significant differences between the AD group and the control samples, the beta diversity analysis based on the Bray–Curtis dissimilarity exhibited distinct clusters based on clinical status, indicating an altered microbiome composition. Specifically, the AD patient group showed a decreased Firmicutes/Bacteroidetes ratio. The Vogt et al. study also highlighted similar trends, noting a shift in the gut microbiota towards reduced diversity but with a heightened relative presence of Bacteroidetes in an observational study involving 25 Alzheimer's patients [10]. This shift, coupled with a probably marked increase in the abundance of Bacteroidetes despite a reduction in their biodiversity, reinforces this microbial group's potential significance in AD pathology [24].

Moreover, the differential abundance analysis highlighted a dysbiosis in the gut microbiota of AD patients, marked by a reduced presence of *Bifidobacterium*, particularly *B. breve*. Such findings align with prior studies that emphasized the positive impact of this bacterial species on cognitive abilities in older adults and observed outcomes in lab animals [25, 26]. For instance, feeding transgenic mice with Bifidobacteria (specifically B. longum 1714) over 6 months resulted in a noticeable decrease in amyloid beta accumulations in the hippocampus and cerebral cortex [27]. Furthermore, research has associated the levels of the primary inhibitory neurotransmitter, gamma-aminobutyric acid (GABA), with the prevalence of Bifidobacteria in the guts of healthy individuals [28]. The altered gut microbiota composition of AD patients in our study notably features an increased presence of pseudomonadota like *Phyllobacterium*, as well as inflammatory bacteria such as Synergistetes (represented by *Cloacibacillus porcorum*) and the Christensenellaceae family. Conversely, beneficial microbes like *Bifidobacterium* declined. Such microbial patterns point to a gut dysbiosis in AD, defined by an upsurge in inflammation-related microbiota and a reduction in microbes that foster health.

The metabolic profiling of the AD microbiome reveals a predominant presence of pathways related to sugar, carrier molecules, tetrapyrrole, pyrimidine biosynthesis, and nucleic acid processing. This analysis also highlighted a possible reduction in carbohydrate, polysaccharide, polyamine, and myo-inositol degradation pathways. When comparing the AD group's gut microbiome to the control group, there was also a reduced representation in pathways such as SCFA fermentation. SCFAs are essential metabolites derived from the fermentation of dietary fiber by the gut microbiota that participate in host metabolism, immune regulation, appetite regulation, etc. Recent studies on gut-brain interaction have shown that SCFAs are important mediators of this axis, and there is evidence that altered SCFA metabolism, particularly butyrate, causescritical biological effects that interfere with the development of AD [29, 30]. The increases in the proinflammatory cytokines IL-1a, IL-8, IL-17A, IL-12p40, TNF-*β*, MCP-1, IL-2, and IL-12p70 and the anti-inflammatory cytokines IL-10 and IL-13 observed in AD patients in our study are consistent with prior evidence of immune dysfunction in AD [31]. Some of these cytokines are also tied to cardiometabolic indicators, probably because such disturbances are connected to Alzheimer's and can exacerbate dementia's advancement [32, 33]. Our analysis pinpointed the inflammatory markers IL-1*α* and IL-8 as top discriminators between AD patients and controls. IL-1*α*, extensively researched for its role in AD, is associated with neuroinflammation and the formation of amyloid beta plaques, hallmarks of AD pathogenesis [31, 34, 35]. IL-8, a chemokine, attracts immune cells like neutrophils during inflammatory events [36, 37]. In AD, IL-8 is also linked to neuroinflammation and may have a neurotoxic effect leading to neuronal death. Clinical studies also support the role of IL-8 in AD [38], with elevated IL-8 concentrations observed in the cerebrospinal fluid of AD patients and individuals with mild cognitive impairment [39], suggesting its relevance in the early phases of the disease.

Notably, in our study, IL-1*α* and IL-8 exhibited negative correlations with various *Bifidobacterium* species. Correlational studies also identified a potential link between elevated cytokine levels in AD patients and a higher prevalence of bacterial groups like Erysipelotrichaceae, Olsenella, and Bacteroidales. Key characteristics pinpointed by the GBDT classification model align with characteristic phenotypic attributes of AD patients, such as high proinflammatory cytokines [40], growth factor dysregulation [41], and gut microbiota imbalance [42]. These findings underscore a potential interplay between specific gut microbes and inflammatory markers. The distinct associations with beneficial *Bifidobacterium* species also hint at potential microbiome-mediated therapeutic avenues.

We have also demonstrated that in AD patients, strong correlations exist between increased serum adiponectin levels and almost all studied cytokines as well as specific microbial taxa, including Actinobacteria, and Acidomicrobiia at the class level, and Prevotella, Faecalibacterium, Christensenellaceae R-7 at the genus level, and Oscillospiraceae at the family level [18]. Adiponectin, a protein hormone primarily synthesized by white adipose tissue, plays a pivotal role in regulating insulin sensitivity, fatty acid metabolism, glucose homeostasis, and the anti-inflammatory response through diverse mechanisms. Adiponectin has been implicated in the pathogenesis of various age-related disorders, including atherosclerosis, type 2 diabetes, cardiovascular ailments, and AD [43]. We have previously reported that serum adiponectin levels were 3-fold higher in the AD group compared to the controls. We have also demonstrated a positive correlation between adiponectin and MMSE scores and high-density lipoprotein cholesterol (HDL-C) in AD patients [43]. Findings of other research groups have found that adiponectin induces the secretion of the anti-inflammatory cytokine IL-10 in lung epithelial A549 cells [44] and stimulates the production of proinflammatory chemokines and cytokines (CXCL1, CXCL5, iIL-6, CCL20, CCL4, CCL3, CCL17, TNF, GM-CSF, CXCL8, CXCL10, CCL5, CCL11, and CCL2) in circulating mononuclear cells and fibroblast-like synoviocytes [45]. Furthermore, data indicates that cross-talks exist between gut microbiota, fatty tissues, and adiponectin expression [46, 47]. This warrants further investigation to elucidate the mechanisms linking the microbiota to AD pathogenesis.

Our study has several notable limitations, primarily related to potential confounding variables, including dietary habits, medication use, and comorbidities, all of which may have some influence on both the gut microbial ecosystem and systemic inflammatory profiles. In addition, the relatively modest size of our sample population and its focus on a specific ethnic cohort limits the broader applicability of our findings. Despite these limitations, our study makes a valuable contribution to the growing body of evidence suggesting a compelling link between AD and alterations in the gut microbiome. These results highlight the importance of further research, with larger, more diverse cohorts and better controls for confounding factors.

## 5. Conclusion

Our findings identify potential microbial and inflammatory signatures in an underrepresented ethnically distinct cohort of AD patients and further support evidence for microbiota-immunity interactions that may influence AD risk. These observed correlations warrant more profound mechanistic studies to determine if gut dysbiosis leads to inflammatory changes that could promote AD pathogenesis, emphasizing the intricate interdependence of immune processes and microbial composition. The data highlight the potential of integrating inflammatory, metabolic, and gut microbial indicators into a signature for differentiating AD. Follow-up analyses should examine links between gut microbes, circulating adiponectin and cytokines, and AD neuropathology, which are needed to establish direct mechanisms.

## Figures and Tables

**Table 1:** Demographic data and clinical characteristics.

**Characteristics**	**Control (** **n** ** = 42)**	**AD (** **n** ** = 41)**	**p** **-value**
Age, *M* ± Sd (IQR)	66.5 ± 9.4 (59.0–73.0)	68.8 ± 9.1 (62.0–74.0)	*p* = 0.28^a^
BMI, *M* ± Sd (IQR)	27.8 ± 4.9 (24.4–30.1)	24.1 ± 3.8 (22.0–24.9)	≤ 0.001^b^
^e^MMSE, *M* ± Sd (IQR)	28.7 ± 1.4 (28.0–30.0)	13.9 ± 8.5 (6.0–22.0)	≤ 0.0001^b^
Adiponectin (ADIPOQ), *M* ± Sd (IQR)	17.8 ± 23.6 (5.7–15.8)	26.6 ± 33.0 (9.8–30.0)	≤ 0.05^b^
Total billirubin (TBIL), *M* ± Sd (IQR)	5.8 ± 2.7 (3.7–7.3)	7.4 ± 3.0 (5.5–8.7)	≤ 0.05^b^
C-reactive protein (CRP), *M* ± Sd (IQR)	7.8 ± 19.4 (1.0–4.6)	8.9 ± 35.1 (0.5–3.6)	*p* = 0.08^b^
Triglycerides (TRIG), *M* ± Sd (IQR)	1.8 ± 1.2 (0.9–2.3)	1.2 ± 0.4 (0.9–1.4)	≤ 0.05^c^
Gender: F/M (%)	35/7 (83.3)	30/11 (73.2)	*p* = 0.3^c^
Self-reported blood pressure: yes/no (%)	24/13 (64.9)	26/13 (66.7)	*p* = 1.0^c^
Self-reported heart disease: yes/no (%)	11/25 (30.6)	12/25 (32.4)	*p* = 1.0^c^
Self-reported stroke: yes/no (%)	3/33 (8.3)	9/29 (23.7)	*p* = 0.1^c^
Self-reported TIA: yes/no (%)	1/34 (2.9)	9/26 (25.7)	≤ 0.05^c^
Self-reported brain injury: yes/no (%)	6/29 (17.1)	4/32 (11.1)	*p* = 0.51^c^
Self-reported diabetes: yes/no (%)	4/33 (10.8)	5/32 (13.5)	*p* = 1.0^c^
Self-reported depression: yes/no (%)	11/25 (30.6)	7/31 (18.4)	*p* = 0.28^c^
Family history of dementia: yes/no (%)	5/29 (14.7)	4/17 (19.0)	*p* = 0.72^c^
Apoliporotein e4 carrier: yes/no (%)	11/24 (31.4)	19/y (50.0)	*p* = 0.15^c^
Ethnicity: Kazakh/Russian/other	30/10/2	30/8/3	*p* = 0.81^d^

Abbreviations: BMI = body mass index; IQR = interquartile range; MMSE = Mini-Mental State Examination; TIA = transient ischemic attack.

^a^Determined by independent T-test.

^b^Determined by Mann–Whitney *U* test.

^c^Determined by Fisher's exact test.

^d^Determined by chi-squared test.

^e^An unauthorized version of the Russian MMSE was used by the study team without prior permission. The MMSE is a copyrighted instrument and may not be used or reproduced in whole or in part, in any form or language, or by any means without written permission of PAR. Retrospective permission to use the collected data for publication was granted by PAR, subject to specific conditions outlined in their letter dated April 22, 2024.

## Data Availability

The datasets presented in this study can be found in online repositories. The names of the repository/repositories and accession number(s) can be found in the following: NCBI BioProject (accession number PRJNA1028813).

## Supporting Information

Supporting Information 1 Figure S1. *α* diversity within the major bacterial phyla indicated by the Shannon indexes.

Supporting Information 2 Figure S2. Ordination using principal coordinate analysis (PCoA) based on Bray–Curtis dissimilarity showing differences in gut microbial composition between groups (beta diversity).
