# Supplementary figures and images for: Inflammatory Manifestations Associated With Gut Dysbiosis in Alzheimer's Disease
Source: Int J Alzheimers Dis. 2024 Sep 20;2024:9741811. doi: 10.1155/2024/9741811 (PMC11436273; doi:10.1155/2024/9741811)

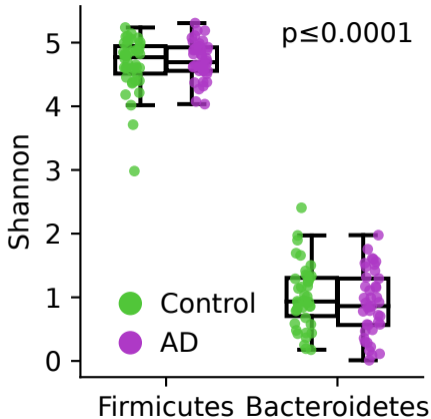

Supplement: Supporting Information 1 — Figure S1. α diversity within the major bacterial phyla indicated by the Shannon indexes. [file 9741811.f1.pdf]

# gut/bc

● control  
● AD

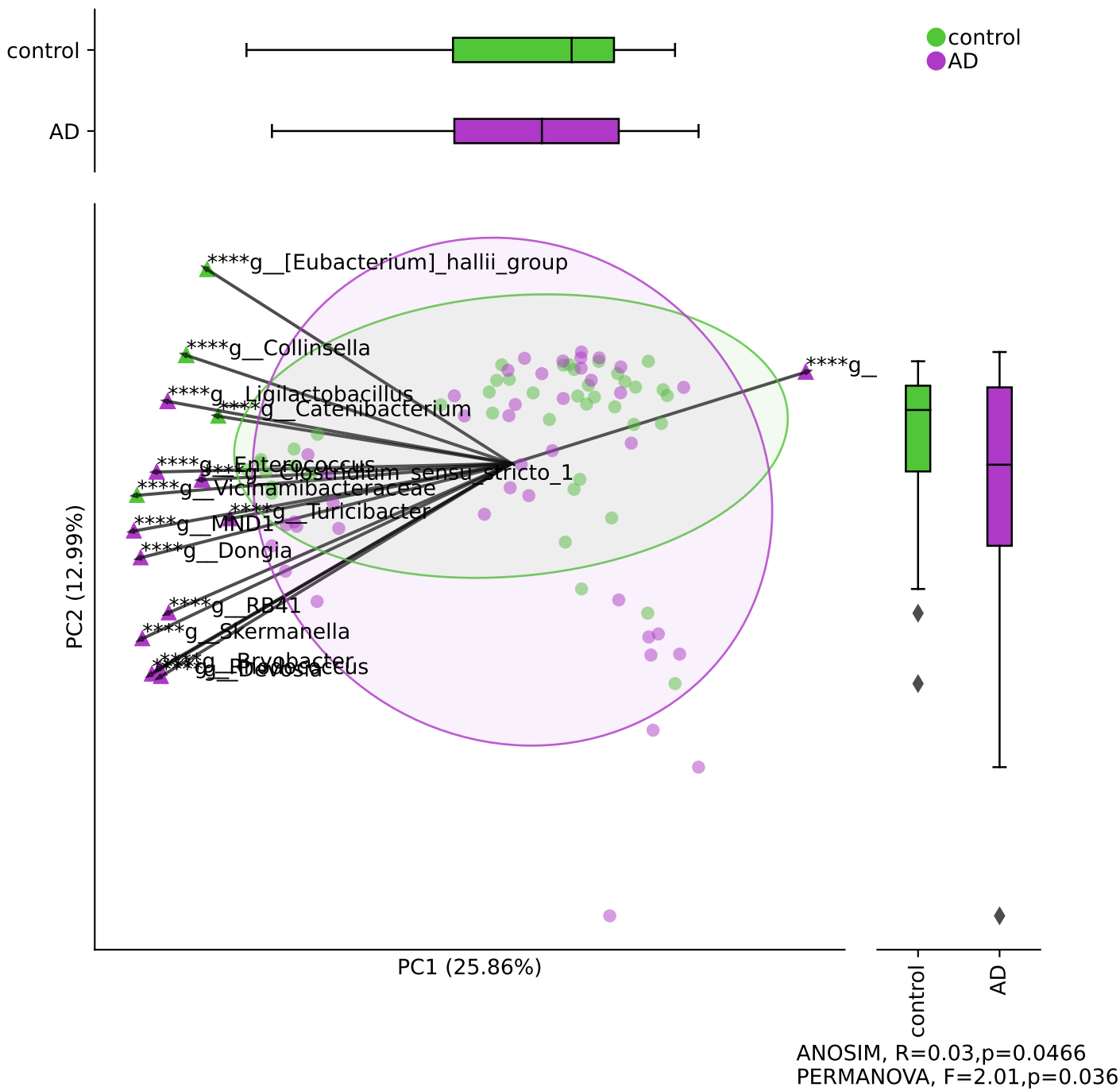

Supplement: Supporting Information 2 — Figure S2. Ordination using principal coordinate analysis (PCoA) based on Bray–Curtis dissimilarity showing differences in gut microbial composition between groups (beta diversity). [file 9741811.f2.pdf]
